# Spontaneous Rupture of a Non-scarring Gravid Uterus: A Late and Haphazard Diagnosis in the Postpartum Period

**DOI:** 10.7759/cureus.66368

**Published:** 2024-08-07

**Authors:** Soufiane Bigi, Mounir Salek, Mohamed Amine Baba, Ahmed Kharbach, Soukaina Wakrim

**Affiliations:** 1 Radiology, Souss Massa University Hospital, Agadir, MAR; 2 Epidemiology and Public Health, Institut Supérieur de Pédagogie et des Sciences de l'Information et des Systèmes de Technologie (ISPITS), Agadir, MAR

**Keywords:** uterine breach, obstetric complication, non-scarring uterus, spontaneous rupture, uterine rupture

## Abstract

This paper reports the case of a spontaneous rupture of a non-scarring gravid uterus seen four days after vaginal delivery and provides an update on this rare pathology, which can be functionally and vitally life-threatening. Uterine rupture of a healthy gravid uterus can occur as a result of structural abnormalities of the uterine tissue framework or uterine parietal fragility due to pathological phenomena such as septic states. On admission, the clinical picture is generally that of an acute abdomen with a hypogastric origin, with or without hemodynamic instability and an altered general condition, depending on the presence of an underlying advanced uterine infection. Medical imaging, mainly ultrasound and CT scan with iodine contrast, enables visualization of the uterine breach and a precise assessment of the damage. Surgery is the treatment of choice for repairing the breach and ensuring hemostasis. This case study sheds light on this pathology, familiarizing us with its clinical and radiological picture, as well as its post-treatment prognosis.

## Introduction

Spontaneous uterine rupture is a rare labor complication in parturient women, posing significant risks to both maternal and fetal outcomes during the peripartum period and even the woman's future fertility if a hysterectomy follows the rupture. The incidence ranges from 1 in 16,840 to 1 in 19,765 births, with fetal mortality rates estimated between 12% and 35% and hysterectomy rates between 20% and 30% [1,2]. This case study aims to review this condition to gather essential insights for investigating and preventing its severe consequences.

## Case presentation

This case is about a 30-year-old woman with no particular pathological history, a mother of two children delivered vaginally with episiotomies repaired without complications. She was admitted postpartum, four days after her second delivery for abdominal symptoms that had been evolving for 24 hours, consisting of painful abdominal bloating with sub-occlusion. Clinical examination revealed generalized abdominal tenderness, predominantly hypogastric, with a fever of 38.2 °C.

The biological assessment showed C-reactive protein at 160 mg/L, hyperleukocytosis at 18,000, neutrophil predominance at around 84%, and correct renal function with blood creatinine at 7 mg/L and blood urea at 0.36 g/L. There was no evidence of anemia (Table 1).

**Table 1:** Patient's biological assessment. Biological inflammatory syndrome without anemia or impaired renal function.

Analysis	Patient value	Normal value
C-reactive protein	160 mg/L	<10 mg/L
Hyperleukocytosis	18,000 mm^-3^	11,000-15,000 mm^-3^
Neutrophil predominance	84%	<40%
Hemoglobin	12.4 g/dL	>12 g/dL
Creatinine	7 mg/L	6-11 mg/L
Urea	0.36 g/L	0.15-0.42 g/L

**Figure 1:**
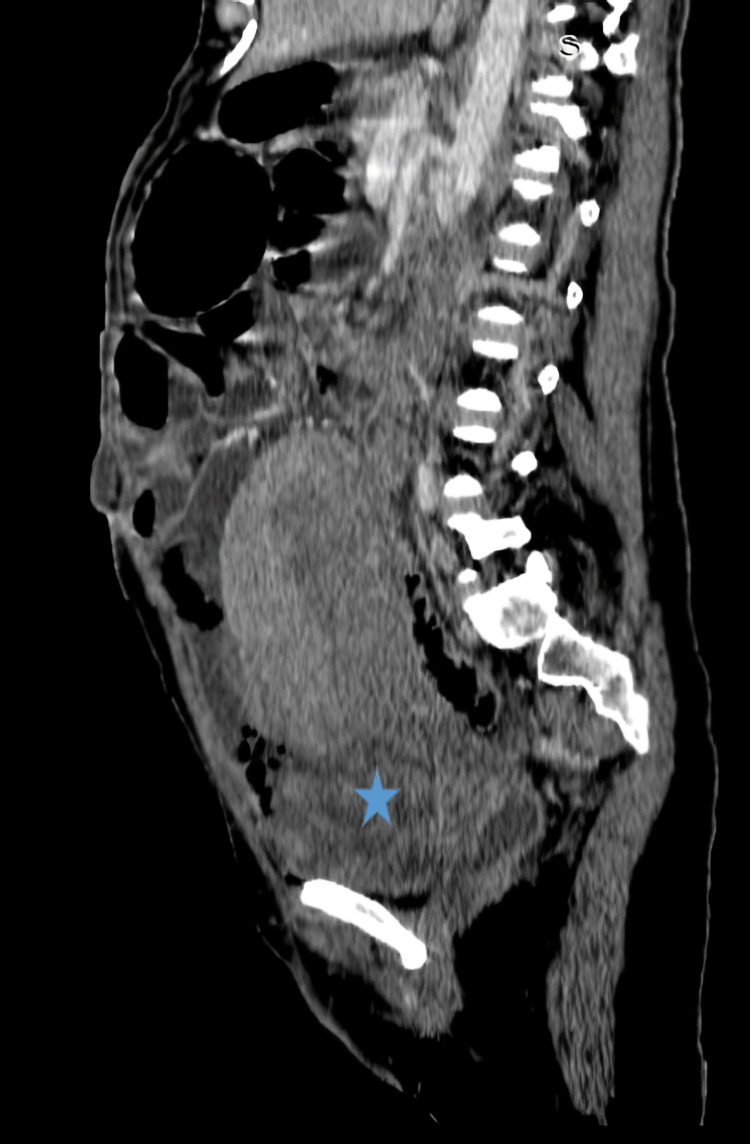
Sagittal scan with iodinated contrast injection. An isthmocorporal uterine parietal breach (star), communicating the uterine cavity with the pelvis.

**Figure 2:**
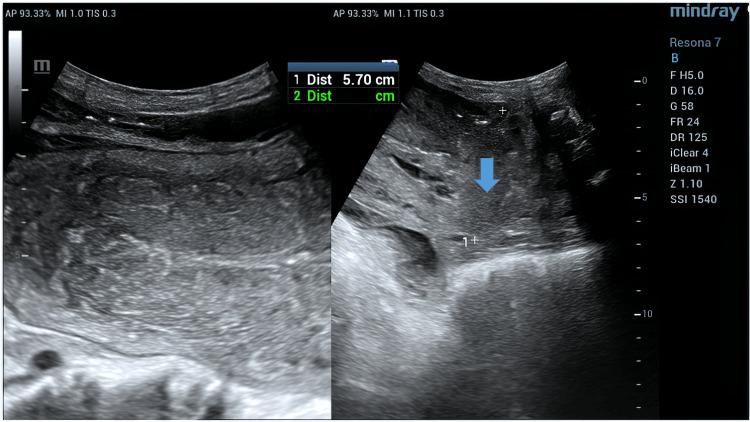
Sagittal pelvic ultrasound slices. An isthmocorporal uterine parietal discontinuity (arrow) communicating the uterine cavity with the pelvis.

An abdominopelvic CT scan with an injection of iodinated contrast was performed on the patient, who presented with an acute post-partum abdomen with sub-occlusion and no post-partum complications, showing an isthmocorporal uterine rupture communicating the uterine cavity with the sub-peritoneal pelvic cavity, with pelvic blood clots and signs of pelvic-peritoneal infection, associated with septic fluid collections containing air bubbles (Figure 1). An additional ultrasound scan was performed to enhance the iconography of the patient's file while the operating room was being prepared for surgery, showing the uterine breach identified on the CT scan (Figure 2).

**Figure 3:**
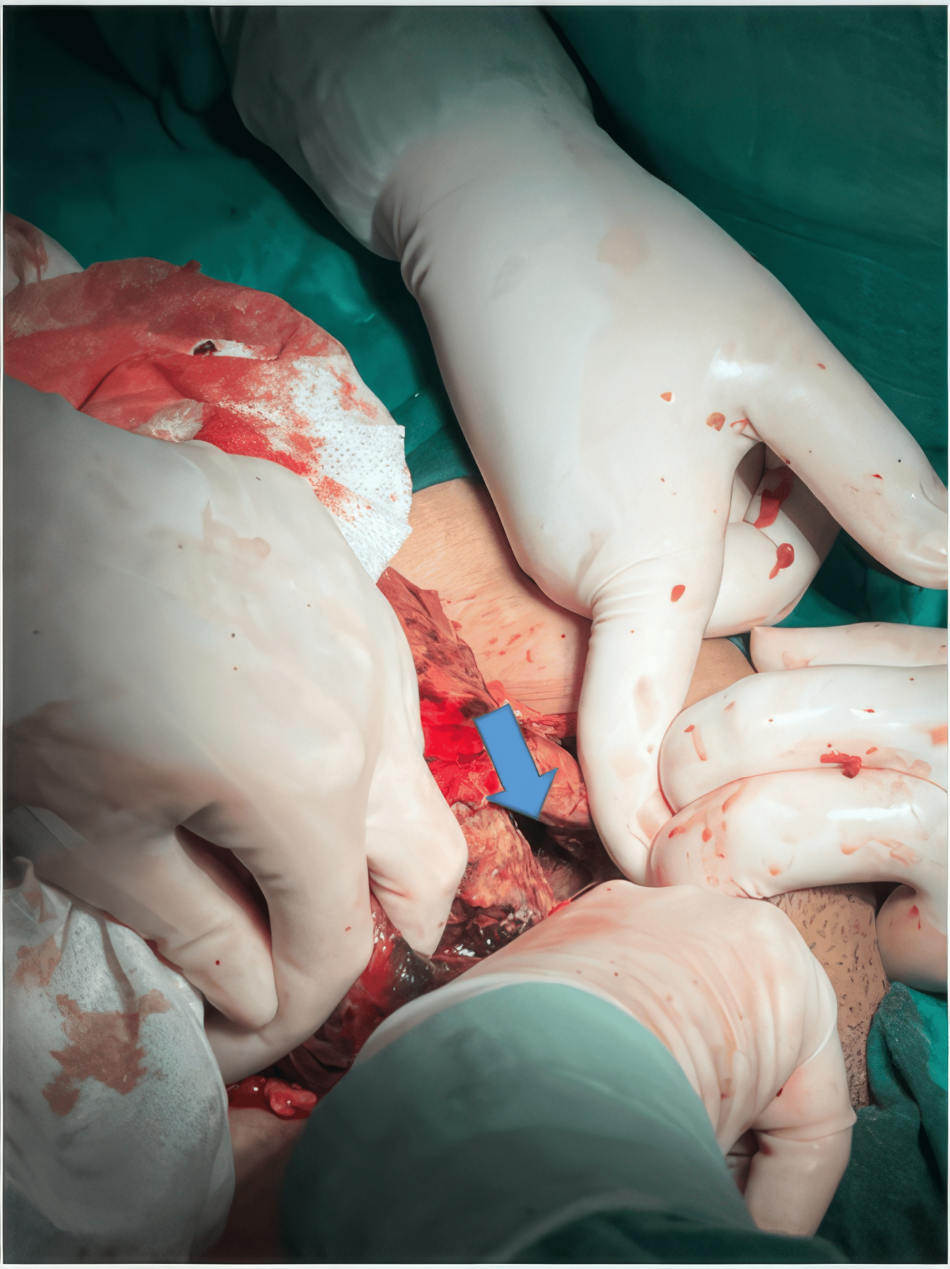
The surgical procedure showing the uterine parietal breach (arrow).

An emergency laparotomy was performed on the young woman, revealing the uterine rupture described on the CT scan (Figure 3), with pelvic hemorrhage and signs of peritonitis. Surgical repair of the uterine breach was performed with hemostasis and peritoneal cleansing, with the placement of a drain to evacuate residual fluids.

The postoperative course was straightforward, with regression of abdominal distension and pain, resumption of transit on the first-day post-surgery, and progressive reduction of the biological inflammatory syndrome under triple broad-spectrum antibiotics.

## Discussion

Uterine rupture in the absence of anterior parietal scarring is much less frequent than that occurring in a post-caesarean scarred uterus. According to recently published studies, the incidence of spontaneous rupture of a gravid uterus is estimated at around 1 per 10,000 pregnancies [3]. Risk factors for this fearsome obstetric complication include multiparity, inappropriate or excessive use of uterotonic drugs, underdiagnosed congenital uterine malformations, as well as invasive obstetric procedures and instrumental extractions [4].

Among the mechanisms underlying spontaneous uterine rupture, intrinsic uterine parietal weakness may be found, in structural abnormalities or also following particular pathological conditions such as intra-uterine infections. Mechanical uterine overload can also be the cause of excessive uterine distension secondary to polyhydramnios or fetal macrosomia [5].

The clinical picture of uterine rupture is that of a painful hypogastrium generalizing into an acute abdomen, bringing together a set of clinical signs evolving in the context of hemodynamic instability, such as sudden and severe abdominal pain, with tenderness or even defensiveness to palpation, associated with fetal heart rate abnormalities. The role of imaging is ultrasound investigating peri-gravidic hypogastric pain in potentially stable patients since the clinic remains sufficient for rapid management of patients received in states of hemodynamic instability [6]. Computed tomography (CT) is indicated in cases where there is no direct ultrasound visualization of the uterine parietal breach, essentially in the case of sub-peritoneal ruptures seen late and blocked by blood clots. Apart from extreme emergencies requiring referral to the operating room for exploration, MRI enables better lesion characterization, a detailed morphological assessment, and the identification of any differential diagnosis other than perforation [7].

The management of a spontaneous non-scarring uterine rupture requires emergency laparotomy, to ensure good hemostasis and repair of the uterine breach, or outright hysterectomy if necessary in the case of difficult hemostasis or extensive necrosis of uterine tissue in the case of late-onset rupture [8]. The prognosis depends on rapid diagnosis and management. A multidisciplinary team, including obstetricians, radiologists, neonatologists, and anesthetists, is often required to optimize management results [9].

Through this case study, which aims to enrich the bibliography, we have shown that it is important to detect uterine ruptures, even in healthy uterus, in cases of risk factors, or the presence of any suggestive clinical sign in the peripartum period, to manage them early rather than seeing them late at the complication stage.

## Conclusions

Spontaneous uterine rupture of a non-scarring uterus is a rare but serious complication of pregnancy. Clinical vigilance is essential to detect uterine rupture and improve its prognosis. Ongoing research is also needed to better understand the pathophysiological mechanisms and develop effective prevention strategies. This would be particularly useful in promoting the health of women of childbearing age and their offspring, to improve health care provision and quality of life in countries, especially in the third world. We also hope that this work will encourage other researchers to investigate this topic in greater depth, in an attempt to gain maximum insight into this public health issue.
